# Pharmacokinetics and pharmacodynamics of an innovative psychedelic N,N-dimethyltryptamine/harmine formulation in healthy participants: a randomized controlled trial

**DOI:** 10.1093/ijnp/pyaf001

**Published:** 2025-01-08

**Authors:** Michael J Mueller, Helena D Aicher, Dario A Dornbierer, Laurenz Marten, Dila Suay, Daniel Meling, Claudius Elsner, Ilhui A Wicki, Jovin Müller, Sandra N Poetzsch, Luzia Caflisch, Alexandra Hempe, Camilla P Steinhart, Maxim Puchkov, Jonas Kost, Hans-Peter Landolt, Erich Seifritz, Boris B Quednow, Milan Scheidegger

**Affiliations:** Department of Adult Psychiatry and Psychotherapy, Psychiatric University Clinic Zurich and University of Zurich, Zurich, Switzerland; Department of Health Science & Technology, ETH Zurich, Zurich, Switzerland; Neuroscience Center Zurich, University of Zurich and Swiss Federal Institute of Technology Zurich, Zurich, Switzerland; Department of Adult Psychiatry and Psychotherapy, Psychiatric University Clinic Zurich and University of Zurich, Zurich, Switzerland; Neuroscience Center Zurich, University of Zurich and Swiss Federal Institute of Technology Zurich, Zurich, Switzerland; Department of Psychology, University of Zurich, Zurich, Switzerland; Department of Adult Psychiatry and Psychotherapy, Psychiatric University Clinic Zurich and University of Zurich, Zurich, Switzerland; Institute of Pharmacology and Toxicology, University of Zurich, Zurich, Switzerland; Department of Forensic Pharmacology and Toxicology, Zurich Institute of Forensic Medicine, University of Zurich, Zurich, Switzerland; Department of Adult Psychiatry and Psychotherapy, Psychiatric University Clinic Zurich and University of Zurich, Zurich, Switzerland; Department of Chemistry and Applied Biosciences, ETH Zurich, Zurich, Switzerland; Department of Adult Psychiatry and Psychotherapy, Psychiatric University Clinic Zurich and University of Zurich, Zurich, Switzerland; IMT School for Advanced Studies, Luca, Italy; Department of Adult Psychiatry and Psychotherapy, Psychiatric University Clinic Zurich and University of Zurich, Zurich, Switzerland; Department of Psychosomatic Medicine and Psychotherapy, Faculty of Medicine, University Medical Center Freiburg, University of Freiburg, Freiburg, Germany; Department of Adult Psychiatry and Psychotherapy, Psychiatric University Clinic Zurich and University of Zurich, Zurich, Switzerland; Department of Adult Psychiatry and Psychotherapy, Psychiatric University Clinic Zurich and University of Zurich, Zurich, Switzerland; Department of Adult Psychiatry and Psychotherapy, Psychiatric University Clinic Zurich and University of Zurich, Zurich, Switzerland; Department of Forensic Pharmacology and Toxicology, Zurich Institute of Forensic Medicine, University of Zurich, Zurich, Switzerland; Department of Adult Psychiatry and Psychotherapy, Psychiatric University Clinic Zurich and University of Zurich, Zurich, Switzerland; Department of Adult Psychiatry and Psychotherapy, Psychiatric University Clinic Zurich and University of Zurich, Zurich, Switzerland; Faculty of Psychology, TU Dresden, Dresden, Germany; Institute of Neuroinformatics, University of Zurich, Zurich, Switzerland; Institute of Pharmaceutical Technology, University of Basel, Basel, Switzerland; Institute of Pharmaceutical Technology, University of Basel, Basel, Switzerland; Department of Adult Psychiatry and Psychotherapy, Psychiatric University Clinic Zurich and University of Zurich, Zurich, Switzerland; Neuroscience Center Zurich, University of Zurich and Swiss Federal Institute of Technology Zurich, Zurich, Switzerland; Institute of Pharmacology and Toxicology, University of Zurich, Zurich, Switzerland; Department of Adult Psychiatry and Psychotherapy, Psychiatric University Clinic Zurich and University of Zurich, Zurich, Switzerland; Neuroscience Center Zurich, University of Zurich and Swiss Federal Institute of Technology Zurich, Zurich, Switzerland; Department of Adult Psychiatry and Psychotherapy, Psychiatric University Clinic Zurich and University of Zurich, Zurich, Switzerland; Department of Adult Psychiatry and Psychotherapy, Psychiatric University Clinic Zurich and University of Zurich, Zurich, Switzerland; Neuroscience Center Zurich, University of Zurich and Swiss Federal Institute of Technology Zurich, Zurich, Switzerland

**Keywords:** pharmacokinetics, pharmacodynamics, psychedelics, N, N-dimethyltryptamine, harmine

## Abstract

**Background:**

Recent interest in the clinical use of psychedelics has highlighted plant-derived medicines like ayahuasca showing rapid-acting and sustainable therapeutic effects in various psychiatric conditions. This traditional Amazonian plant decoction contains N,N-dimethyltryptamine (DMT) and β-carboline alkaloids such as harmine. However, its use is often accompanied by distressing effects like nausea, vomiting, and intense hallucinations, possibly due to complex pharmacokinetic/pharmacodynamic (PK-PD) interactions and lack of dose standardization.

**Methods:**

This study addresses these limitations by testing a novel pharmaceutical formulation containing pure forms of DMT and harmine in a double-blind, randomized, placebo-controlled trial with 31 healthy male volunteers. We evaluated PK-PD by monitoring drug and metabolite plasma levels, subjective effects, adverse events, and cardiovascular parameters. Each participant received 3 randomized treatments: (1) 100 mg buccal harmine with 100 mg intranasal DMT, (2) 100 mg buccal harmine with intranasal placebo, and (3) full placebo, using a repeated-intermittent dosing scheme, such that 10 mg of DMT (or placebo) was administered every 15 minutes.

**Results:**

N,N-dimethyltryptamine produced consistent PK profiles with C_max_ values of 22.1 ng/mL and acute drug effects resembling the psychological effects of ayahuasca with a duration of 2–3 hours. Likewise, buccal harmine produced sustained-release PK profiles with C_max_ values of 32.5 ng/mL but lacked distinguishable subjective effects compared to placebo. All drug conditions were safe and well tolerated, indicating the formulation’s suitability for clinical applications.

**Conclusions:**

This study underscores the potential of a patient-oriented pharmaceutical formulation of DMT and harmine to reduce risks and improve therapeutic outcomes in treating mental health disorders.

**Clinical trial registration number:**

Neurodynamics of prosocial emotional processing following serotonergic stimulation with N,N-dimethyltryptamine (DMT) and harmine in healthy subjects (NCT04716335) https://clinicaltrials.gov/ct2/show/NCT04716335

**Significance Statement**

Our research provides important insights into the pharmacokinetics (PK) and pharmacodynamics (PD) of a novel N,N-dimethyltryptamine (DMT)/harmine formulation designed to improve individualization (ie, flexible administration protocol), feasibility (ie, short duration), safety, and tolerability in psychedelic-based treatments for mental health. Traditional ayahuasca, which contains these compounds, has shown potential therapeutic effects but is often accompanied by significant adverse reactions and intense experiences. In this study, we tested a new formulation of intranasal DMT and buccal harmine in healthy participants, using a repeated-intermittent dosing approach to achieve sustained drug release. Our findings demonstrated favorable PK/PD profiles, with good tolerability and few side effects. This study represents a step forward in developing safe, controllable psychedelic therapies for individualized mental health care.

## INTRODUCTION

Psychedelic compounds such as psilocybin, lysergic acid diethylamide (LSD), and N,N-dimethyltryptamine (DMT) are currently experiencing renewed interest as potential treatments for various mental health disorders.^1^ The indigenous Amazonian plant brew “ayahuasca” containing the potent psychedelic compound DMT has become the focus of international research endeavors.^2-5^ DMT is increasingly being recognized as a promising drug candidate given its distinct effects on serotonergic homeostasis and neuroplasticity compared to conventional serotonergic antidepressants.^6,7^ Converging lines of evidence from preclinical and human studies suggest that oral formulations containing DMT such as ayahuasca are associated with substantial reductions in anxiety and depressive symptoms.^2,5^ Notably, ayahuasca shows equally rapid but more sustainable antidepressant effects than ketamine.^2,8-10^

While the exact pharmacological mechanisms have not yet been fully revealed, a drug-drug interaction between the indole alkaloid DMT and the β-carboline alkaloids harmine, harmaline, and tetrahydroharmine is likely mediating its therapeutic effects. DMT shows no oral bioavailability due to excessive metabolic first-pass degradation by the enteric monoamine oxidase A (MAO-A) enzyme.^11^ Thus, herbal sources of DMT (eg, from *Psychotria viridis*) are frequently combined with β-carboline-containing plants (eg, from *Banisteriopsis caapi*), serving as selective and reversible MAO-A inhibitors, ensuring reduced first-pass metabolism and prolonged duration of DMT action.^12^ DMT interacts primarily with 5HT2A, 5HT2C, 5HT1A, and sigma receptors,^7,13^ leading to widespread changes in neuronal excitability with pronounced effects on mood, perception, and cognition.^14^ While the effects of intravenous bolus DMT are characterized by their rapid onset and experiential intensity,^15-17^ orally administered DMT in the form of ayahuasca evokes a state of introspective awareness, dream-like visions, intensified emotions, and autobiographical memories, lasting up to 4-6 hours.^18,19^ DMT exhibits acute, short-term tolerance, with a nonlinear relationship between plasma levels and subjective effects during successive administrations within a short period. When an additional dose is administered during the acute phase, response intensity and plasma concentration do not increase proportionally. However, as DMT is rapidly metabolized with rapidly declining acute effects, subsequent dosing can elicit effects similar to the initial dose. However, the mechanisms underlying the rapid reversal of tolerance to DMT compared to other psychedelics such as psilocybin, LSD, and mescaline, which can take several days, remain to be further explored.^7,20-30^

Although ayahuasca is considered safe when administered in a controlled setting,^18,19,31^ herbal ayahuasca can induce distressing somatic and psychological effects including strong nausea, vomiting, diarrhea, and overwhelming perceptive and psychological sensations.^32^ While from the indigenous perspective, these effects are considered key therapeutic factors, they may limit the clinical applicability of ayahuasca in vulnerable patient populations. A previous study comparing different DMT- and harmine-containing formulations suggested that some of the ayahuasca-like intolerabilities may be related to unpredictable pharmacokinetic and pharmacodynamic (PK-PD) profiles of the alkaloid mixtures,^33^ where variations in absorption, metabolism, and receptor response may result in inconsistent drug effects, potentially leading to overdosing and suboptimal drug-drug interactions. In particular, bypassing the gastrointestinal (GI) tract through administering harmine via the oromucosal and DMT via the intranasal route was found to substantially reduce GI-related side effects, as well as metabolic first-pass-related PK variabilities. Harmine was chosen due to better tolerability compared to harmaline and due to more potent MAO inhibition compared to tetrahydroharmine.^34^

Given these promising findings, the present work further investigates the clinical pharmacology of combined oromucosal harmine and intranasal DMT administration in 31 healthy male volunteers, using a randomized, placebo-controlled, crossover design. To evaluate the PD of combined DMT/harmine and harmine alone, all volunteers participated in 3 separate drug conditions: (1) 100 mg buccal harmine plus 100 mg intranasal DMT, (2) 100 mg buccal harmine plus intranasal placebo, and (3) buccal placebo plus intranasal placebo. DMT applications followed a repeated-intermittent dosing regimen, in which 10 mg were administered every 15 minutes. PK profiles of DMT (plus its metabolites N-methyltryptamine [NMT], DMT-N-oxide, and indole-3-acetic acid [3-IAA]) and harmine (plus its metabolite harmol) were assessed by means of continuous blood sampling. Moreover, vital signs and acute psychometric variables were assessed regularly.

In sum, this study aimed at exploring the clinical pharmacological profile of a combined parenteral DMT/harmine formulation, thus contributing to the understanding of its mechanisms of action and clinical translation potential.

## METHODS

### Participants and Permission

Out of 37 healthy male volunteers, a total of 31 participants (25.4 ± 4.2 years) with a mean body mass index (BMI) of 23.0 ± 1.9 SD participated in all 3 study days and experienced all 3 different conditions. Out of the 6 dropouts (27.4 ± 5.7 SD years), 4 volunteers dropped out before study day 1, and 2 volunteers dropped out after day 1 because of personal reasons. The following criteria were required for inclusion: male sex in order to avoid the potential impact of menstrual cycle on repeated endocrinological measures; age within the range of 20 to 40 years; BMI between 18.5 and 30; no current or previous history of somatic, neurological, or psychiatric disorder according to case history and Structural Clinical Interviews for DSM (SCID-I; SCID-II); no family history of Axis-I psychiatric disorders; no acute or chronic medication intake; and no current drug use, no or little history of psychedelic experience (eg, LSD, psilocybin, ayahuasca, etc. ^35^). The study was approved by the Cantonal Ethics Committee of the Canton of Zurich (Basec-Nr. 2018-01385) and the Swiss Federal Office of Public Health (BAG-Nr. [AB]-8/5-BetmG-2019/008014). All participants provided written informed consent according to the declaration of Helsinki. All participants received monetary compensation.

### Study Setting

The study was carried out during the daytime at the University of Zurich. Rooms were climate-controlled with a cozy living room environment and fitted with adjustable lighting and sound systems. A standard playlist of background music was played throughout the study day. Participants were seated comfortably on a mattress leaning against the wall and supported by cushions. An experimenter was present at all times to oversee the participants.

### Study Design

**Figure 1.**
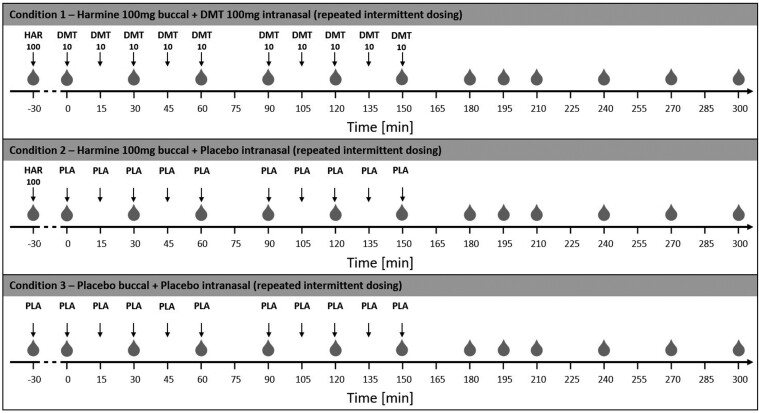
Illustration of the study procedure. Time points of blood withdrawal are indicated as gray drops (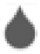). On all study days, harmine (or corresponding placebo) was applied 30 minutes before the first DMT (or corresponding placebo) dosing, to provide sufficient MAO inhibition at the time point of DMT administration. Harmine (or corresponding placebo) was given buccally, whereas DMT (or corresponding placebo) was administered intranasally in a repeated-intermittent manner. Thereby, volunteers were allowed to discontinue the DMT administration in case of tolerability issues or adverse effects. At each administering time point, either a dose of 0, 5, or 10 mg could be selected. Nevertheless, volunteers were motivated to stick to the original dosing protocol if they felt well. DMT, N,N-dimethyltryptamine; MAO, monoamine oxidase.

The study was conducted as a within-subject, double-blind, randomized placebo-controlled trial. Randomization was performed by an independent individual using a computer-generated random number sequence. All participants underwent 3 randomized drug treatments on separate study days, with an intervening washout period of at least 2 weeks: (1) DMT/HAR: harmine hydrochloride (HCl; 100 mg; buccal orodispersible tablet; ODT) plus DMT (as hemifumarate; 100 mg; intranasal: 10 mg every 15 minutes over 150 minutes), (2) HAR/PLA: harmine (100 mg, buccal ODT) plus placebo nasal spray (NaCl; at same dosing intervals), and (3) PLA/PLA: placebo buccal ODT plus placebo nasal spray. In all conditions, buccal tablets were premedicated for 30 minutes. Food or water intake was not allowed 2 hours before drug administration. The detailed dosing scheme is depicted in Figure 1.

### Study Drug

DMT hemifumarate (98.2% purity) was extracted and purified from Mimosa hostilis root bark (The Mimosa Company). Harmine hydrochloride (≥98% purity) was sourced from Santa Cruz Biotechnology Inc. Preparation, Analytical Procedures, and Stability Testing are described in the Supplementary Materials (SM).

### Intranasal DMT Formulation

DMT hemifumarate was aseptically dissolved in NaCl 0.9% to form a nasal spray solution with a concentration of 2.5 mg per puff. The solution was then transferred into nasal spray pump systems with a puff volume of 50 μL (Aptar Pharma) containing a total of 100 mg (+ 20% excess) of DMT. The 20% excess volume was added to avoid aspiration of air and consequently dilution of the administered dose.

### Buccal Harmine Formulation

Harmine HCl ODTs for buccal delivery were obtained by freeze-drying. Therefore, harmine HCl, mannitol, and hydroxypropyl methylcellulose were dissolved in deionized water, volumetrically filled into aluminum blister molds, and freeze-dried for 30 hours. For each study day, one ODT containing 100 mg of harmine was manufactured.

### Intranasal Placebo Formulation

A fumaric acid (1% in NaCl 0.9%) was aseptically manufactured and transferred into nasal spray pump systems with a puff volume of 50 μL (Aptar Pharma).

### Buccal Placebo Formulation

Dextran-based ODTs were obtained by freeze-drying. Therefore, Dextran was dissolved in deionized water, volumetrically filled into aluminum blister molds, and freeze-dried for 30 hours.

### Dose Regimen

Thirty minutes following buccal premedication with harmine HCl (100 mg) or placebo, the intranasal repeated-intermittent administration of DMT or placebo was initiated. Volunteers received up to 100 mg DMT in 10 mg dosing steps each 15 minutes (2 × 2 puffs per nostril) over a period of 150 minutes (except for a 30-minute break between 60 and 90 minutes due to a behavioral task). Doses were defined based on our previous study.^33^ Volunteers could adjust each dose (0, 5, 10 mg) based on tolerance, with encouragement to follow the regimen if no adverse effects (AEs) occurred. Thus, volunteers were given the chance to control the psychedelic strength of the experience to enhance safety and tolerability. Only 2 participants skipped a dose, the option of 5 mg was never chosen. See Figure 1 for the dosing schedule.

### Blood Sampling

Blood samples were collected from the left or right antecubital vein at −30 (baseline), 0, 30, 60, 90, 120, 150, 180, 195, 210, 240, 270, and 300 minutes after first DMT/placebo administration for analysis of blood plasma levels of harmine, DMT, the 3 major DMT metabolites 3-IAA, DMT-N-oxide, and NMT and the major harmine metabolite harmol. While DMT administration was ongoing, blood drawing was always performed right before DMT administration (time point -1 minute). The venous catheter was connected to a 100 mm Heidelberger plastic tube extension to collect blood samples without disturbing the volunteers during their psychedelic experience. The intravenous line was kept patent with a slow drip (10 mL/h) of heparinized saline (1000 IU heparin in 0.9 g NaCl/dL; HEPARIN Bichsel; Bichsel AG). Blood samples were immediately centrifuged for 10 minutes at 2000 RCF. Then, plasma was transferred to Eppendorf tubes, shock-frosted in liquid nitrogen (~ −196 °C) and stored at −80 °C until assay. An overview of the blood withdrawal time points is visualized in Figure 1.

### Analysis of Blood Levels

Can be found in SM.

### Psychometry

The intensity of acute subjective effects was monitored with visual analog scales (VASs, range 0–100) on a touchscreen tablet throughout the study day at baseline (−90), 0, 30, 60, 90, 120, 180, 240, and 300 after the first DMT/placebo administration. At 360 minutes after the first DMT/placebo administration, a phenomenological interview was conducted with participants to further explore acute subjective drug effects. For PK-PD analyses, we included VAS for intensity, liking, disliking, and arousal in the present paper. Further analyses on a set of neurophysiological, psychometric, and phenomenological assessments will be presented in forthcoming manuscripts. ^35^

### Vital Signs and AEs

The participants were monitored with regard to AEs throughout the experiment by the study physician, including questionnaire-based assessments (VAS, 0-100 or y/n) of physical and mental discomfort, breathing difficulties, racing heartbeat, chest or abdominal pains, unpleasant body sensations/muscle pain, headache, nausea, vomiting, and fainting at baseline (−105), 0, 30, 60, 120, 240, and 300 minutes after DMT/placebo administration. Vital signs (systolic/diastolic blood pressure [DBP], heart rate, body temperature [BT]) were assessed using a semiautomatic blood pressure and oral temperature recording device throughout the study at baseline (−45), 0, 30, 120, 150, 210, and 270 minutes after the first DMT/placebo administration.

### Statistical Analysis

The data were analyzed and visualized with R Studio version 2021.09.2 + 382.^36^ PK-PD parameters were computed using the R-package PKNCA.^37^ For C_max_, t_max_, AUCs (area under the curves), and half-life calculations, non-compartmental analyses were performed.^38^ According to the results of a Shapiro test (assumption test), non-parametric Friedman tests were used to compare drug conditions. Post hoc pairwise comparisons were calculated using paired Wilcoxon signed-rank tests. *P*-values were adjusted using the Benjamini-Hochberg multiple testing correction method. Pearson or Spearman correlations were performed (as appropriate according to Shapiro-Wilk tests) to analyze associations between DMT plasma levels and subjective effects.

## RESULTS

### Pharmacokinetics

**Figure 2.**
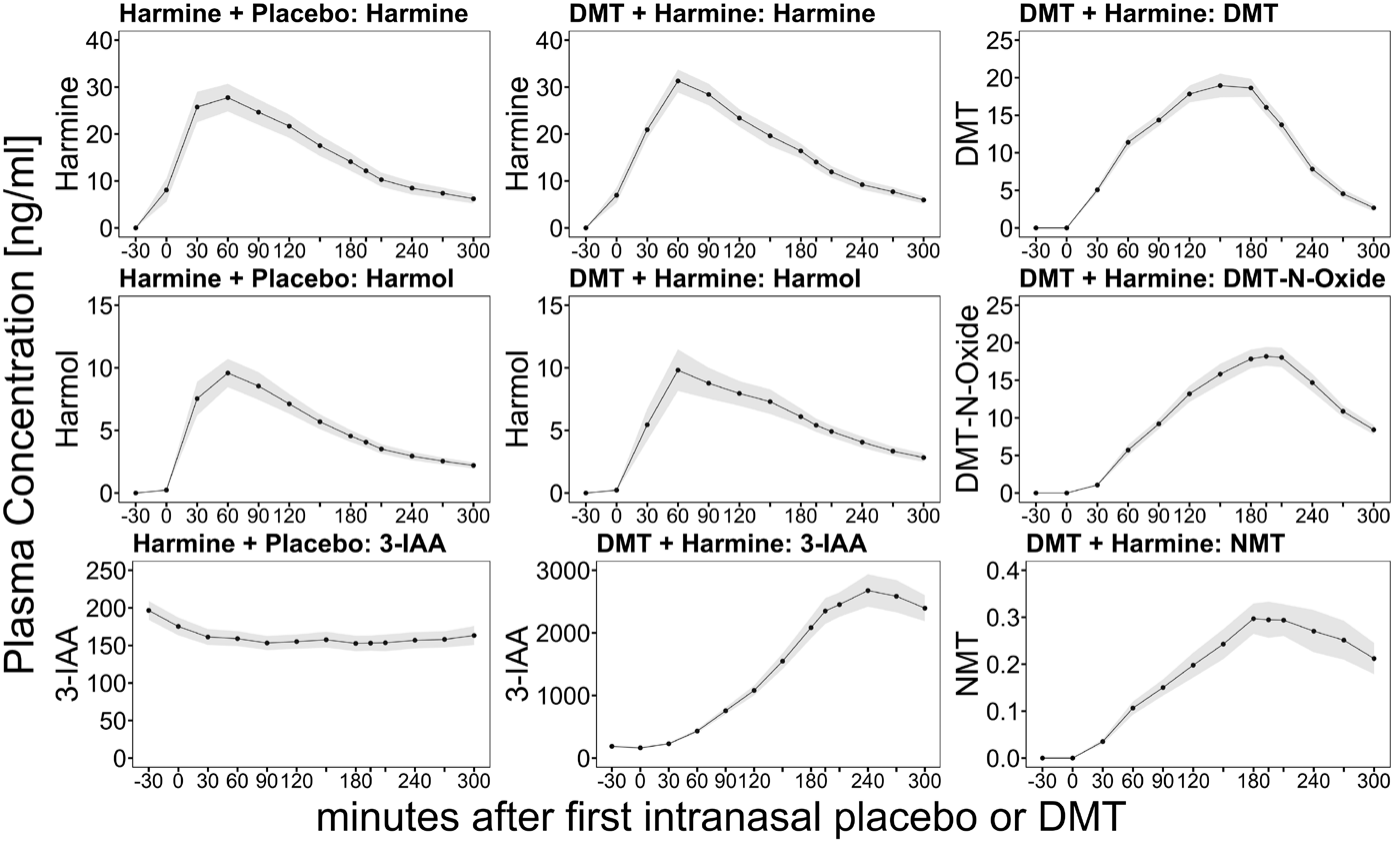
Time course of the blood plasma profiles of DMT and harmine (first row), DMT-N-oxide and harmol (second row), and NMT and 3-IAA (third row) after the administration of harmine + placebo (first column) and DMT + harmine (second and third column). The lines indicate mean analyte concentrations (displayed in ng/mL), and shades indicate the SEM. The *x*-axis displays the time (minutes) in relation to the start of the intranasal placebo/DMT administration at time point 0. 3-IAA, indole-3-acetic acid; DMT, N,N-dimethyltryptamine.

The time course of the mean plasma concentrations of the study compounds DMT and harmine, as well as DMT metabolites, namely 3-IAA, DMT-N-Oxide, NMT, and the harmine metabolite harmol are shown in Figure 2. The corresponding pharmacokinetic parameters are listed in Table 1.

**Table 1.** Pharmacokinetic parameters for N,N-DMT and its metabolites, and harmine and its metabolite in the DMT + harmine condition and harmine + placebo condition based on non-compartmental analyses.

Condition marker	C_max_ (ng/mL)	t_max_ (h)	t_1/2_ (h)	AUC_all_ (ng × h/mL)	AUC_inf_ (ng × h/mL)
DMT + harmine					
DMT	22.1 (7.1)	2.7 (0.5)	0.6 (0.3)	56.6 (17.3)	59.9 (19.4)
DMT-N-oxide	21.0 (7.0)	3.2 (0.6)	1.2 (0.4)	55.4 (17.8)	69.2 (22.1)
NMT	0.4 (0.3)	3.3 (0.7)	2.4 (2.4)	1.0 (0.6)	1.6 (1.6)
3-IAA	2912.2 (1442.8)	4.1 (0.6)	-	7578.7 (3250.2)	-
Harmine	32.5 (12.2)	1.6 (0.6)	1.4 (0.7)	89.1 (36.1)	104.1 (48.0)
Harmole	12.0 (8.3)	1.6 (1.2)	1.8 (1.4)	29.5 (17.6)	36.8 (20.6)
Harmine + placebo					
Harmine	33.5 (17.6)	1.4 (0.4)	1.5 (0.8)	83.5 (47.7)	99.5 (63.8)
Harmole	12.5 (7.5)	1.6 (0.6)	2.0 (0.8)	26.4 (13.2)	32.6 (15.6)

Means and SDs (displayed in brackets) are shown. Temporal values refer to the administration time of DMT or harmine.

Abbreviations: AUC_all_, area under the plasma concentration-time curve from time zero to last time point; AUC_inf_, area under the plasma concentration-time curve from time zero to infinity; C_max_, estimated maximum plasma concentration; DMT, N,N-dimethyltryptamine; 3-IAA, indole-3-acetic acid; NMT, N-methyltryptamine; t_1/2‐_, estimated plasma elimination half-life; t_max_, estimated time to reach C_max_.

For each study day with DMT plus harmine (DMT/HAR) and harmine plus placebo (HAR), blood samples from 29 out of 31 participants could be analyzed. Exclusions from the plasma analysis were due to failure of sampling blood caused by cannula dislocation (1 case in DMT/HAR; 2 cases in HAR) or accidental swallowing of the harmine ODT (1 case in DMT/HAR).

In the DMT/HAR condition, the mean peak plasma concentration (C_max_) of DMT was 22.1 ng/mL (7.1 SD). The time point with the maximum plasma concentration (t_max_) of DMT was reached 2.7 hours (0.5 SD) after the initiation of repeated-intermittent intranasal DMT administration over a period of 150 minutes. We determined a mean elimination half-life (t_1/2_) of 0.6 hours (0.3 SD) for DMT (when co-administered with harmine), with an AUC_inf_ of 59.9 ng × h/mL (19.4 SD). The mean peak plasma concentration (C_max_) of harmine was 32.5 ng/mL (12.2 SD). The time point with the maximum plasma concentration (t_max_) of harmine was reached 1.6 hours (0.6 SD) after harmine ODT administration. The mean elimination half-life (t_1/2_) of harmine was 1.4 hours (0.6 SD), with an AUC_inf_ of 104.1 ng × h/mL (48.0 SD). The pharmacokinetic parameters of the metabolites of DMT and harmine are shown in Table 1.

In the HAR condition, we found a comparable C_max_ of 33.5 ng/mL (17.6) for harmine, and t_max_ was reached 1.4 hours (0.4 SD) after harmine administration with a mean t_1/2_ of 1.5 hours (0.8 SD) and AUC_inf_ of 99.5 ng × h/mL (63.8 SD). 3-IAA levels decreased after harmine administration. The pharmacokinetic parameters of the metabolite of harmine are shown in Table 1.

The blood plasma profiles of DMT-N-oxide (metabolite of DMT) and harmole (metabolite of harmine) followed the profiles of their precursors with a slight time delay. In contrast, the blood plasma levels of the other DMT metabolites, namely NMT and 3-IAA, showed a more sustained increase and longer lasting plateau, which corresponds to a longer elimination half-life. Notably, we observed a disproportional increase in the plasma concentrations of 3-IAA compared to the other metabolites.

In some participants, the blood concentration of some metabolites was not back to baseline at the later blood drawings, and therefore the elimination half-life (t_1/2_) and AUC_inf_ of the metabolites NMT, 3-IAA, and DMT-N-oxide could not be calculated for all participants. This was most prominent for the metabolite 3-IAA, due to its prolonged elimination half-life, t_1/2_, and the AUC_inf_ was only measurable in 5 participants; therefore, we do not report any mean values here. For NMT and DMT-N-oxide, t_1/2_ and AUC_inf_ were measurable in 16 and 24 participants, respectively.

### Pharmacodynamics

#### Subjective Effects

— Compared to placebo (PLA) or harmine alone (HAR), combined DMT/harmine (DMT/HAR) administration induced significant alterations in the state of consciousness with psychedelic effects lasting up to 240 minutes (max. intensity ratings at 90 minutes: contrast DMT/HAR—HAR: effect size: 0.808; p_adj._ < 0.001; contrast DMT/HAR—PLA: effect size: 0.847, p_adj,_ < 0.001; last significant difference at 240 minutes: contrast DMT/HAR—HAR: effect size 0.359, p_adj._ = 0.014; contrast DMT/HAR—PLA: effect size 0.407, p_adj._ = 0.014). We found no statistical differences in subjective ratings of acute drug effects between the harmine alone and placebo condition (max. intensity ratings at 90 minutes: contrast HAR-PLA effect size 0.133, p_adj._ = 0.307; 240 minutes: contrast HAR-PLA: effect size 0.127, p_adj._ = 1).

**Figure 3.**
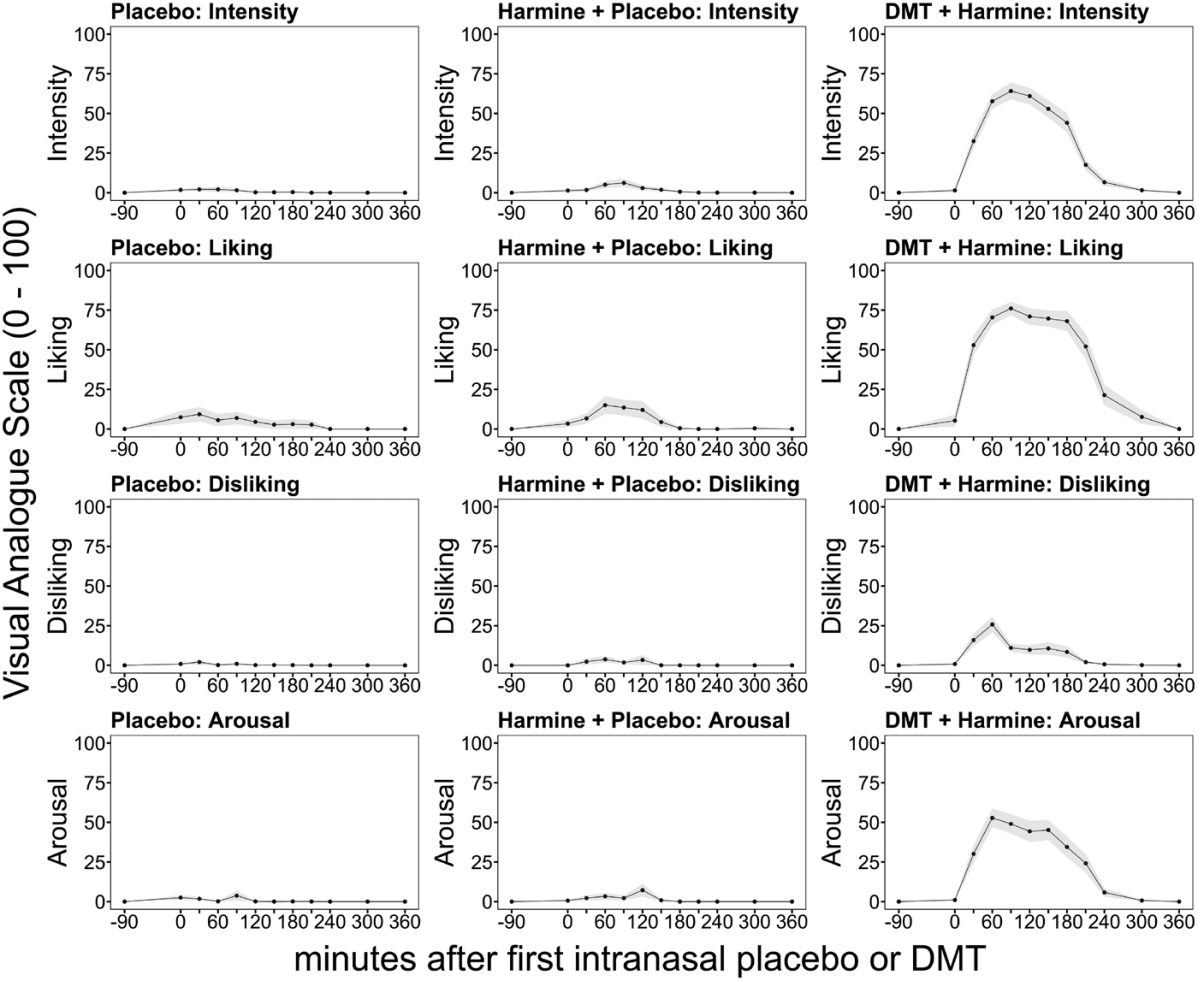
Subjective drug effects for intensity (first row), liking (second row), disliking (third row), and arousal (fourth row) for placebo + placebo (first column), harmine + placebo (second column), and DMT + harmine (third column). Black lines indicate mean ratings on visual analogue scales (VAS; 0-100), and gray shades indicate the SEM. The *x*-axis displays the time (minutes) related to the start of intranasal placebo/DMT administration at time point 0. DMT, N,N-dimethyltryptamine.

In Figure 3, acute subjective drug effects including (1) intensity, (2) liking, (3) disliking, and (4) arousal are shown as VASs at different time points throughout the study days. DMT/HAR induced robust increases in subjective intensity, liking, and arousal. Transient disliking was reported in some volunteers mostly during the first hour and is likely associated with the onset of acute DMT effects, which may be distressing to drug-naïve participants, particularly while performing neurobehavioral tasks. The corresponding peak effects were reported around 90 minutes after the initiation of repeated-intermittent intranasal DMT administration over 150 minutes, with a lasting plateau until 180 minutes, followed by a rapid decrease of subjective effects. Reported peak intensity reached an average of 75.9% (22.2 SD) of the maximal possible score in DMT/HAR, considerably higher than in HAR (7.6% [15.9 SD]). Similarly, reported peak effects of liking reached an average of 88.0% (14.7 SD), considerably higher than in HAR (20.0% [35.2 SD]). Comparably lower levels of disliking were reported with mean peak effects of 41.7% (28.9 SD) in DMT/HAR, considerably higher than in HAR (7.8% [21.9 SD]). Reported peak effects of arousal reached an average of 66.2% (30.2 SD) in DMT/HAR, considerably higher than in HAR (9.9% [23.1 SD]). Besides a slight increase in liking with a reported peak effect reaching on average 11.0% (28.5 SD) of the maximal possible score, there were no notable changes in PLA.

The estimated time to peak effects after drug administration were all in the range between 60 and 120 minutes for both DMT/HAR and HAR, regardless of the subjective effect.

Acute subjective effects gradually subsided between 180 and 240 minutes after the first DMT administration, but mild afterglow effects were still reported in the qualitative interviews taken after the last assessment around 360 minutes after drug administration. Most subjective effects (except disliking) approximately followed the blood plasma concentrations of DMT, indicating that intranasal DMT is efficiently activated by buccal harmine. We observed a strong positive correlation between subjective intensity ratings and DMT plasma levels (*r* = 0.59, 95% CI, 0.51-0.66, *t*(305) = 12.68, *P* < .001).

#### Vital Signs

**Figure 4.**
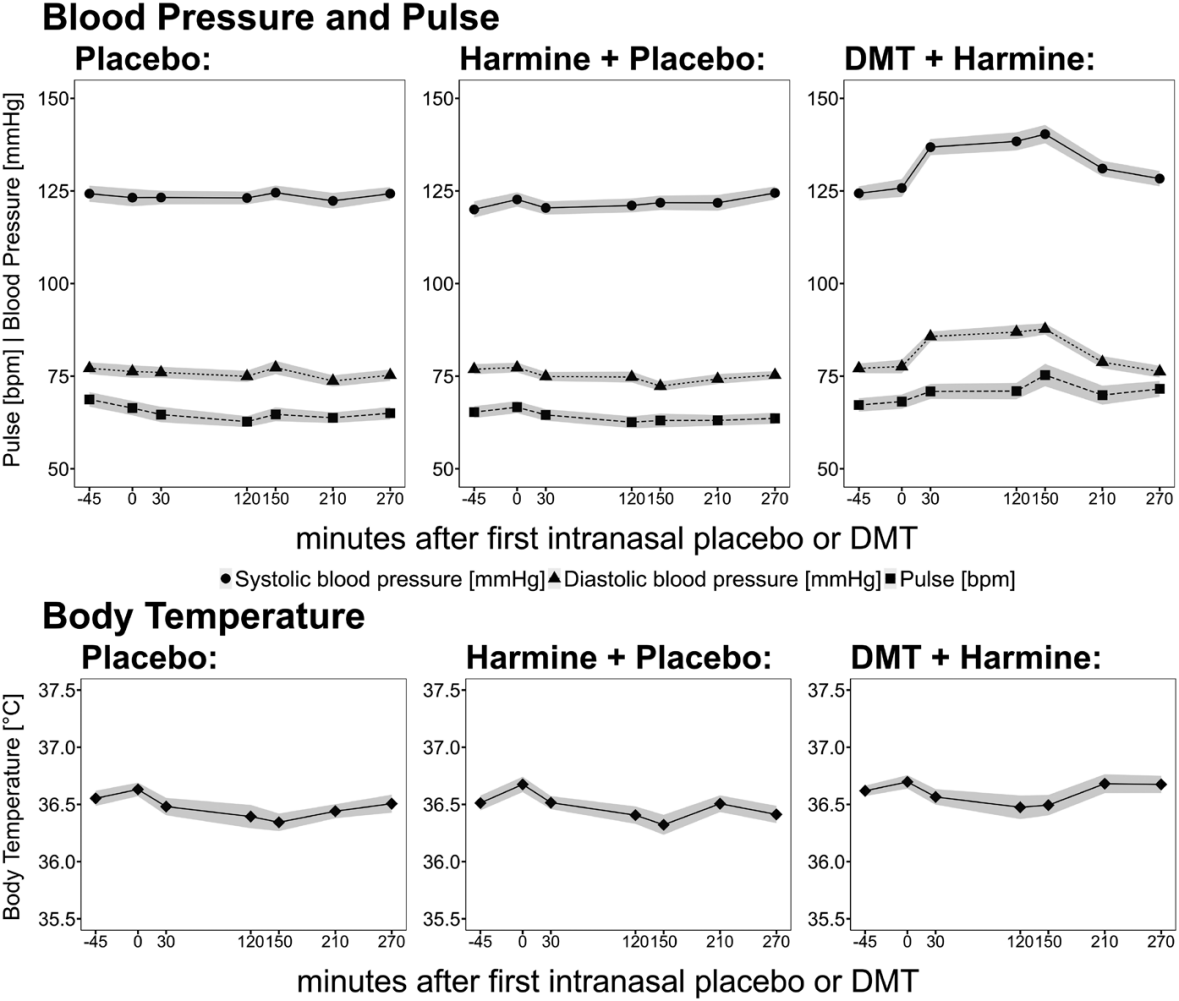
Vital signs (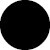 systolic BP; 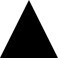 diastolic BP, ■ pulse, 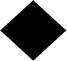 body temperature) during pharmacological challenge with placebo (left) vs harmine (middle) vs intranasal DMT/buccal harmine (right). Black lines indicate mean values, and gray shades indicate the SEM. The *x*-axis displays the time (minutes), based on the start of intranasal placebo/DMT administration at time point 0. BP, blood pressure; DMT, N,N-dimethyltryptamine.

— Mean values for BT, systolic blood pressure (SBP), DBP, and heart rate over time are shown in Figure 4. DMT/HAR induced low to moderate elevations of the cardiovascular parameters, and no relevant changes were observed in HAR, nor in PLA. Mean heart rate values stayed in the range of 60 to 75 bpm. SBP increased on average by 16.5 (9.9 SD) mmHg, and DBP increased by 10.3 (10.2 SD) mmHg, with peak values at 120-150 minutes after initial DMT administration (values are likely confounded by neurobehavioral tasks). No relevant change was observed in BT.

#### Undesired Drug Effects

— In Table 2, the number of participants reporting AEs are shown for all drug conditions. Most AEs were observed in the DMT/HAR condition during the first hour when subjective drug effects increased. Around 30–40% of participants experienced transient AEs such as moderate levels of nausea (*n* = 9/31; mean intensity 46.9/100), heart racing (*n* = 12/31; mean intensity 29.8/100), and somatic distress (*n* = 14/31; mean intensity 26.1/100), which resolved rapidly and did not require medical intervention. Compared to the background noise in the reporting of AEs (eg, placebo condition), the drug-induced AEs for both HAR and combined DMT/HAR can be considered as mild and well tolerated.

**Table 2.** Number of participants reporting undesired drug effects with y/n and visual analog scales, means of intensity [0–100], and SDs (displayed in brackets) across all participants are reported for the 3-drug conditions (placebo, top panel; 100 mg oromucosal harmine, medium panel; 100 mg oromucosal harmine with 100 mg repeated-intermittent DMT; bottom).

	Minutes after first intranasal DMT/placebo administration						
	−105	0	60	120	180	240	300
**Placebo**							
Somatic distress	9; 13 (12)	8; 12 (11)	5; 21 (23)	4; 22 (25)	5; 20 (15)	6; 26 (15)	5; 18 (12)
Psychological distress	1; 11 (0)	2; 2 (2)	2; 14 (15)	0; 0 (0)	1; 8 (0)	2; 7 (3)	0; 0 (0)
Breathing difficulty	0; 0 (0)	2; 2 (1)	1; 2 (0)	3; 1 (1)	0; 0 (0)	1; 1 (0)	0; 0 (0)
Heart racing	2; 10 (3)	2; 1 (0)	1; 4 (0)	0; 0 (0)	2; 35 (45)	1; 54 (0)	2; 2 (1)
Chest pain	1; 3 (0)	0; 0 (0)	0; 0 (0)	1; 1 (0)	0; 0 (0)	0; 0 (0)	0; 0 (0)
Stomach ache	4; 28 (38)	3; 23 (34)	0; 0 (0)	0; 0 (0)	2; 33 (42)	0; 0 (0)	0; 0 (0)
Muscle ache	6; 21 (23)	4; 13 (9)	3; 18 (7)	4; 16 (12)	4; 14 (9)	4; 22 (10)	3; 14 (9)
Head ache	2; 3 (0)	5; 18 (13)	7; 12 (19)	4; 42 (35)	4; 28 (23)	6; 23 (12)	3; 29 (13)
Nausea	2; 4 (4)	3; 5 (4)	3; 7 (5)	1; 2 (0)	1; 5 (0)	0; 0 (0)	0; 0 (0)
Vomiting	0; 0 (0)	0; 0 (0)	0; 0 (0)	0; 0 (0)	0; 0 (0)	0; 0 (0)	0; 0 (0)
Fainting	0; 0 (0)	0; 0 (0)	0; 0 (0)	0; 0 (0)	0; 0 (0)	0; 0 (0)	0; 0 (0)
**Harmine**							
Somatic distress	7; 17 (12)	5; 18 (14)	5; 9 (5)	7; 7 (5)	4; 13 (3)	4; 16 (12)	3; 12 (5)
Psychological distress	4; 13 (8)	3; 6 (2)	2; 8 (8)	2; 4 (5)	1; 9 (0)	2; 3 (1)	1; 70 (0)
Breathing difficulty	2; 12 (15)	3; 18 (25)	2; 21 (28)	3; 7 (8)	0; 0 (0)	1; 6 (0)	1; 3 (0)
Heart racing	3; 9 (6)	2; 28 (35)	1; 9 (0)	3; 5 (5)	1; 1 (0)	1; 3 (0)	1; 49 (0)
Chest pain	0; 0 (0)	2; 24 (32)	2; 13 (8)	3; 4 (3)	0; 0 (0)	1; 1 (0)	0; 0 (0)
Stomach ache	2; 12 (15)	1; 2 (0)	1; 52 (0)	2; 30 (37)	0; 0 (0)	2; 32 (44)	1; 2 (0)
Muscle ache	5; 11 (9)	6; 9 (7)	2; 22 (0)	3; 10 (9)	4; 16 (14)	3; 21 (5)	2; 9 (11)
Head ache	3; 8 (7)	1; 3 (0)	2; 34 (47)	1; 11 (0)	1; 72 (0)	1; 27 (0)	0; 0 (0)
Nausea	1; 6 (0)	1; 2 (0)	3; 11 (10)	1; 15 (0)	1; 1 (0)	1; 2 (0)	2; 2 (1)
Vomiting	0; 0 (0)	0; 0 (0)	0; 0 (0)	0; 0 (0)	0; 0 (0)	0; 0 (0)	0; 0 (0)
Fainting	0; 0 (0)	0; 0 (0)	0; 0 (0)	0; 0 (0)	0; 0 (0)	0; 0 (0)	0; 0 (0)
**DMT + Harmine**							
Somatic distress	9; 13 (11)	7; 11 (5)	14; 26 (24)	11; 14 (12)	7; 25 (15)	3; 6 (3)	1; 37 (0)
Psychological distress	6; 9 (5)	3; 4 (4)	11; 9 (8)	5; 16 (11)	5; 32 (20)	1; 14 (0)	0; 0 (0)
Breathing difficulty	4; 6 (5)	3; 10 (8)	10; 19 (27)	3; 16 (21)	3; 12 (16)	1; 7 (0)	1; 4 (0)
Heart racing	2; 16 (8)	2; 17 (21)	12; 30 (26)	5; 17 (17)	6; 24 (18)	1; 1 (0)	2; 2 (2)
Chest pain	0; 0 (0)	2; 4 (0)	4; 24 (39)	4; 6 (6)	0; 0 (0)	1; 2 (0)	1; 2 (0)
Stomach ache	0; 0 (0)	5; 18 (21)	0; 0 (0)	4; 9 (13)	0; 0 (0)	0; 0 (0)	1; 4 (0)
Muscle ache	5; 20 (9)	8; 27 (24)	4; 34 (37)	5; 12 (9)	3; 22 (12)	2; 12 (16)	1; 3 (0)
Head ache	2; 6 (7)	6; 8 (4)	4; 15 (20)	1; 8 (0)	0; 0 (0)	4; 15 (10)	0; 0 (0)
Nausea	0; 0 (0)	2; 10 (5)	9; 47 (26)	5; 29 (33)	3; 43 (22)	2; 48 (64)	0; 0 (0)
Vomiting	0; 0 (0)	0; 0 (0)	1; 1 (0)	0; 0 (0)	0; 0 (0)	0; 0 (0)	0; 0 (0)
Fainting	0; 0 (0)	0; 0 (0)	0; 0 (0)	0; 0 (0)	0; 0 (0)	0; 0 (0)	0; 0 (0)

Values greater than zero are highlighted in gray.

Abbreviation: DMT, N,N-dimethyltryptamine.

## DISCUSSION

The study examined an innovative ayahuasca-inspired formulation—comprising a synergistic combination of DMT and harmine—in 31 healthy male volunteers. By means of pharmaceutical reformulation, the study aimed at mitigating pharmacological challenges associated with oral DMT preparations (such as herbal ayahuasca or pharmahuasca), including inter-/intrasubject variability in bioavailability and metabolism, and gastrointestinal side effects.^27,32^ The developed pharmaceutical formula entailed the buccal premedication of a harmine ODT, followed by repeated-intermittent intranasal dosing of DMT over 150 minutes. To evaluate for the first time the pharmacological contribution of harmine to the overall subjective experience, this study compared the psychological effects of harmine and co-administration of harmine plus DMT.

DMT is one of the few short-acting psychedelics that has recently gained interest for the treatment of psychiatric and neurological conditions. DMT is not orally bioavailable due to excessive first-pass metabolism by the MAO enzyme system.^11^ In contrast, parenteral DMT administrations by means of inhalation or intravenous bolus infusion yield high systemic exposure levels, followed by rapid elimination within minutes (t_1/2_ between 5 and 16 minutes).^13,17^ However, the effects of DMT can be significantly potentiated and prolonged by reducing its metabolic breakdown through the concurrent inhibition of the MAO enzyme system.^12,39^ This concept is the foundation of the indigenous Amazonian plant medicine ayahuasca, which combines plant-based sources of DMT with harmala alkaloid-rich plants, acting as selective and reversible MAO-A inhibitors. More recently, synthetic or purified sources of DMT and harmine have been used to create standardized versions of ayahuasca, also known as ayahuasca-analogue or pharmahuasca.^40^ While the synergistic principle underlying the combination of MAO inhibitors (eg, harmala alkaloids) and DMT has been proven to work, several PK-PD challenges limit the usability of such preparations for pharmacotherapeutic purposes. In particular, dose-exposure relationship of ayahuasca was found to vary dramatically across individuals, which may be attributed to differences in gastrointestinal absorption and first-pass metabolism. Consequently, difficulties standardizing the dose and the risk of overdosing may be associated with some of the frequently reported AEs in traditional ayahuasca settings.^32^ Thus, we hypothesized that optimizing dose-exposure predictability represents the first step toward a safe use of oral DMT formulations.

The GI tract and first-pass metabolism represent the major source of pharmacokinetic variations across individuals.^41^ To this end, in this study, both harmine and DMT were pharmaceutically delivered in a parenteral fashion. In this sense and in line with PK data from a previous study,^33^ we found that harmine was well absorbed buccally and produced blood plasma levels with low interindividual variability, in contrast to oral ayahuasca.^19^ Interestingly, the buccal route of administration favored a sustained-release profile, which appeared to be particularly suited for the extended DMT administration protocol employed in this study.

Premedication of harmine significantly increased the elimination half-life of DMT (t_1/2_ of 36 minutes) compared to other studies administering DMT only,^17^ enabling continuous intermittent dosing of DMT over 150 minutes, resulting in an overall drug action of up to approx. 240 minutes. Unlike bolus IV injection, inhaled, or single oral administrations (ayahuasca/pharmahuasca), the repeated-intermittent dosing of DMT yielded a sustained-release PK profile with a pronounced plateau. After the last intranasal DMT dose, blood plasma levels remained constant for 30 minutes before starting to decrease. This is in strong contrast to inhaled or intravenous bolus DMT, which results in short-lasting high-plasma DMT peaks.^15^ However, when DMT is administered as continuous IV infusion, it can also produce more stable prolonged plateau concentrations.^17^ The remaining DMT blood plasma levels at 210 minutes post-administration in our study indicate that residual levels of MAO inhibition of harmine are still in effect. Moreover, by bypassing the first-pass metabolism through buccal administration, lower doses of harmine seem to be sufficient to prevent rapid clearance of DMT by MAO and to extend the psychedelic experience compared to traditional ayahuasca.^42^ In our investigation, the decision to use a lower harmine dosage was driven by the intention to minimize side effects. However, the optimal dose level of buccal harmine in combination with DMT still needs to be determined. Therefore, while our results are promising, they underscore the necessity for systematic dose-response studies of combined DMT/harmine formulations to pinpoint the optimal dosage that induces the strongest therapeutic effects while minimizing side effects.

The ratio between a parent substance (DMT and harmine) and its main metabolite (3-IAA/N-oxide DMT and harmol, respectively) enables the estimation of the location of absorption. In more detail, oral administrations followed by excessive first-pass metabolism yield smaller ratios (higher metabolite level) compared to parenteral administration followed by slow systemic metabolism. Indeed, we found considerably higher harmine:harmol ratios (~3:1) compared to traditional ayahuasca, where in some cases even ratios below 1 were found (no detectable harmine levels^18^). For DMT, we found that—despite co-administration of harmine—the majority was degraded to 3-IAA by MAO-A.^17,43^ Anyhow, DMT-N-oxide plasma levels were significantly higher compared to plasma levels obtained after ayahuasca or pure DMT (i.v., smoked) administration.^17,34^ This may indicate a slight shift from MAO-A to cytochrome P450 mediated degradation in the presence of an MAO inhibitor.^43^ In the present study, DMT was still predominantly metabolized via the MAO system, indicating that the MAO system was by far not completely blocked (but rather slowed down) in the measured harmine exposure range. However, the combination of serotonergic drugs such as SSRIs with MAO inhibitors remains contraindicated due to the potential risk of serotonin syndrome, as emphasized in current treatment guidelines. Although observational studies on ayahuasca consumption have shown no statistically significant difference in AEs between participants using antidepressants and those who did not,^44^ the safety and risks of drug-drug interactions involving combined harmine/DMT preparations require further investigation.

Moreover, we found that 3-IAA (which is not only the main DMT metabolite but also an endogenous metabolite of the amino acid tryptophan) levels were decreased in the harmine-only condition, indicating a harmine-related decrease in tryptophan breakdown to 3-IAA by MAO.^45-47^ This effect may account for some of the harmine’s indirect neuromodulating effects, given the role of tryptophan in the biosynthesis of serotonin and other neuroactive molecules.

In line with previous research, the subjective effects of the present DMT/harmine formulation were mainly driven by DMT. We found no significant difference in subjective ratings between HAR and PLA, suggesting that harmine at the administered dose strength acts primarily as a PK-enhancer of DMT. However, we observed a slight increase in liking, which may be in line with previously reported relaxing properties of harmine.^48,49^ Furthermore, the administered harmine dose and consequently the exposure levels were rather low in this study, compared to ayahuasca administrations reported in the past.^50^ Thus, it cannot be excluded that harmine (and other harmala alkaloids) may have PD contributing effects at higher doses. DMT increases proprioceptive sensitivity, which may potentiate the sensation of subjective harmine effects. Also, the isolated effects of DMT were not examined in this study, such that pharmacodynamic contributions of harmine to the overall experience cannot be fully elucidated with the data at hand.

Overall, participants responded very well to the parenteral delivery of DMT, with high levels of intensity and liking as well as moderate arousal and comparably low and transient levels of disliking. Interestingly, the peak of the subjective effects slightly proceeded the peak plasma concentration of DMT and reached plateau levels despite continued DMT administration and still increasing plasma levels. A similar effect was observed with intravenous DMT.^17^ While we found a clear exposure-effect relationship, the initial doses produced stronger changes in subjective rating compared to later dose increments. Thus, changes from baseline (ordinary consciousness) may be perceived as stronger than changes occurring in a plateau. As such, bolus administrations of DMT (eg, intravenous and inhaled) can rapidly perturb neurochemical homeostasis and may thus bear a pronounced risk potential compared to slow-release formulations or dosing protocols.^13,51^ Thus, in this study, intranasal DMT was dosed incrementally in 15-minute intervals over 150 minutes, which resulted in robust DMT exposure levels, closely corresponding to the subjective drug effects. Given the high degree of controllability of this approach, the dosing format was highly appreciated by all study volunteers and may also increase patient compliance in clinical settings.

While the reported undesired drug effects (such as mild levels of somatic or psychological distress, dizziness, headache, nausea, increased heart rate, and blood pressure; Table 2, Figure 4) were similar in nature compared to ayahuasca typical side effects,^32^ the frequency and intensity of AEs in the present study were considerably lower (eg, nausea/vomiting: 29% of participants compared to 62% of traditional ayahuasca users). In some religious ayahuasca settings, vomiting can be as frequent as 97% of users,^44^ compared to 3% of participants in our study. It is assumed that this amelioration of AE intensity may be driven by reduced stimulation of serotonergic chemoreceptors in the GI tract, due to non-peroral dosing. It must be mentioned that some of the AEs observed in this study could have been potentiated by the fact that participants were engaging in several neurobehavioral tasks (at 60, 150, and 210 minutes), posing additional challenges, specifically under the influence of a psychedelic. Furthermore, some reported AEs (eg, headache, muscle ache) were even higher in the placebo (PLA) condition than in the HAR or DMT/HAR condition, possibly due to the study setup (EEG recordings). Overall, the AEs for both HAR and DMT/HAR were mild and well tolerated. Noteworthy, we observed a mild but asymptomatic increase in systolic and DBP and heart rate in the DMT/HAR condition, which is in line with previous studies.^11,15,17,50^ The increase in cardiovascular activity may also have been confounded by some behavioral tasks (time window 120–150 minutes) that may have enhanced psychophysiological arousal levels. Ayahuasca as an extract of numerous plants contains a highly complex mixture of alkaloids, which makes it difficult to attribute pharmacological or AEs to any of the ingredients or microbiological contaminations. In contrast, the present formula formulation is fully standardized and by circumventing the GI tract, the predictability and tolerability of drug effects are further optimized (eg, by reducing individual differences in GI absorption and hepatic first-pass metabolism, and by sparing intestinal 5-HT3 chemoreceptors on vagal afferent terminals).

Although there are many differences between botanical ayahuasca and DMT/harmine formulations, which obviously limits their direct comparison, we noticed a striking similarity in the reported subjective effects (eg, intensity, liking, and duration).^18^ Moreover, the beneficial AE profile and potentially improved PK-PD of this DMT/harmine formulation^33^ may have clear advantages for clinical applications, as growing evidence shows that ayahuasca and DMT-based formulations have the potential to improve mental health.^3-5,9^ However, further studies are needed to directly compare ayahuasca and DMT/harmine formulations. Although the combined administration of DMT and harmine is more complex, it is likely to yield more favorable experience profiles to support psychotherapy compared to DMT alone.^35^ Given the need to reduce the psychological risks associated with the use of psychedelic drugs, this patient-controlled administration of a psychedelic substance is novel and seems particularly beneficial for future applications in the treatment of psychiatric patients. In sum, compared to the clinical data on traditional ayahuasca^32^, the clinical pharmacological profile might be improved by means of pharmaceutical reformulation.^33^

It is important to consider several limitations when interpreting the results of this study. Although double-blinded, randomized, placebo-controlled designs are widely used in therapeutic trials, they have limitations for the investigation of psychedelics. The potent and distinct effects of DMT potentially reduce the impact of a double-blinded randomization due to expectancy and reporting biases,^52^ especially affecting self-report measures. Moreover, contextual setting variables such as behavioral tasks or music may significantly impact the overall subjective experience. In addition, we deliberately chose moderate dose levels to enable participation in behavioral experiments. To explore the full pharmacological range of effects of DMT/harmine, further studies should explore various dose combinations of DMT and harmine including DMT alone and harmine alone. Finally, the study sample consisted of Caucasian, male, young, and average-weight individuals, thus not allowing for translation of the results to a more heterogeneous population. Further research with a more diverse sample is needed to better ascertain the safety, tolerability, and efficacy of this intervention.

## CONCLUSION

We here present the PK-PD properties of a novel combined parental administration of DMT as a nasal spray and harmine as an ODT in a first double-blind, randomized, placebo-controlled, within-subject study in healthy male volunteers. Our PK-PD results are consistent with the pharmacological principle of herbal ayahuasca, wherein the rapid degradation of DMT in the body is slowed down by co-administration with the MAO-A inhibitor harmine, thereby prolonging DMT’s psychedelic effects for the duration of 2-3 hours. Moreover, our results indicate that subjective psychedelic effects were primarily driven by DMT. Repeated-intermittent dosing of DMT further improves safety and tolerability compared to oral ayahuasca preparations and may represent a more reliable and controllable solution for administering DMT/harmine in clinical trials. The commonly reported psychosomatic side effects and distress with traditional oral ayahuasca could be strongly attenuated via parenteral administration bypassing the gastrointestinal tract. This novel approach appears to be valuable for clinical applications especially in vulnerable patient populations due to the improvement of dosing uncertainty and unpredictable side effects associated with the administration of psychedelic compounds. We conclude that combined intranasal DMT and buccal harmine may become an innovative rapid-acting, safe, and patient-oriented treatment solution for affective disorders.

## Supplementary Material

pyaf001_suppl_Supplementary_Materials

## Data Availability

The data supporting the findings of this study are not publicly available due to sensitivity and confidentiality considerations. Anonymized raw data may be made available upon reasonable request from the corresponding author, subject to approval by the University of Zurich.
