# Supplementary material for: Pharmacokinetics and pharmacodynamics of an innovative psychedelic N,N-dimethyltryptamine/harmine formulation in healthy participants: a randomized controlled trial
Source: Int J Neuropsychopharmacol. 2025 Jan 8;28(1):pyaf001. doi: 10.1093/ijnp/pyaf001 (PMC11770821; doi:10.1093/ijnp/pyaf001)
Supplement: pyaf001_suppl_Supplementary_Materials [file pyaf001_suppl_supplementary_materials.docx]

**Supplementary Material**

***Study drug.*** DMT hemifumarate was obtained by acidic-basic aqueous extraction from the root bark of Mimosa hostilis (The Mimosa Company, 1069CL Amsterdam, NL), with n-heptane as organic solvent. DMT was purified by crystallisation and further recrystallized as DMT hemifumarate via salt precipitation. This was then dried under vacuum. The final product was subjected to qualitative and quantitative analysis via quantitative Nuclear Magnetic Resonance (qNMR), liquid chromatography-tandem mass spectrometry (LC-MS/MS), and high-performance liquid chromatography (HPLC), revealing a purity of 98.20% ± 0.37%. Harmine hydrochloride (Harmine HCl, ≥98% HPLC-tested) was procured from Santa Cruz Biotechnology Inc. (Dallas, Texas 75220, USA) and further purified via basic precipitation, recrystallization and HCl precipitation. DMT hemifumarate for the nasal spray solution was weighed and checked according to the four-eye principle. Content uniformity of the harmine buccal tablets was confirmed via HPLC. Chemical stability of harmine and DMT hemifumarate was demonstrated in forced degradation studies following GMP guidelines. While stability of the DMT nasal sprays was not assessed in this study, stability of DMT hemifumarate solutions has been confirmed elsewhere (Vogt et al., 2023). For the buccal tablets, stability has been assumed due to the high chemical stability of harmine observed under all forced degradation conditions and the chemical inertness of the used excipients (mannitol and HPMC). In addition, the study medication was stored dark at 20°C for a short duration until completion of the study (4 months).

***Analysis of blood levels.*** DMT was purchased from Lipomed (Arlesheim, Switzerland), NMT and 3-IAA were purchased from Sigma-Aldrich (St. Louis, USA), and harmine, harmol, DMT-N-oxide, harmine-d3 and DMT-d6 were purchased from Toronto Research Chemicals (Toronto, Canada). All other used chemicals were of highest grade available.

For the sample preparation 200 µl of plasma were spiked with 50 µl internal standard (IS) mixture (40 ng/ml DMT-d6 and harmine-d3) and 50 µl methanol (MeOH). Proteins were precipitated by adding 400 µl of acetonitrile (ACN). The samples were shaken for 10 minutes and centrifuged for 5 min at 10‘000 rpm. 350 µl of the supernatant was transferred into an auto-sampler vial, evaporated to dryness under a gentle stream of nitrogen at room temperature and reconstituted in 100 µl eluent-mixture (98:2, v/v). External calibrator and quality control (QC) samples were prepared accordingly, replacing the MeOH with calibrator or QC solution mixtures. Calibrator and QC samples containing 3-IAA were prepared separately, replacing plasma by water. The calibration ranges were 0.5–500 ng/ml for DMT and DMT-N-oxide, 2.5–120 ng/ml for harmine, 1–80 ng/ml for harmol, 0.015–10 ng/ml for NMT and 35–3000 ng/ml for 3-IAA.

Samples were analysed on an ultra-high performance liquid chromatography (UHPLC) system (Thermo Fisher, San Jose, CA) coupled to a linear ion trap quadrupole mass spectrometer 5500 (Sciex, Darmstadt, Germany). The mobile phases consisted of a mixture of water (eluent A) and ACN (eluent B), both containing 0.1% formic acid (v/v). Using a Kinetex C18 column 50 × 2.1 mm, 2.6 µm (Phenomenex, Aschaffenburg, Germany), the flow rate was set to 0.5 ml/min with the following gradient: starting conditions 98% eluent A, decreasing to 70% within 4 min, followed by a quick decrease to 5% within 1 min, holding for 0.5 min and returning to starting conditions for 1.5 min, resulting in a total runtime of 7 min. The mass spectrometer was operated in positive electrospray ionization mode with scheduled multiple reaction monitoring. The following transitions of precursor ions to product ions were selected as quantifier ions: DMT m/z 189→115, DMT-N-oxide m/z 205→117, harmine m/z 213→169, harmol m/z 199→131, NMT m/z 175→144 and 3-IAA m/z 176→103. The HPLC method has been validated according to GLP standards and will be presented elsewhere.

**References**

Vogt SB, Ley L, Erne L, et al. Acute effects of intravenous DMT in a randomized placebo-controlled study in healthy participants. Transl Psychiatry. 2023;13(1):1-9. doi:10.1038/s41398-023-02477-4
